# Exercise protects against diabetic cardiomyopathy by the inhibition of the endoplasmic reticulum stress pathway in rats

**DOI:** 10.1002/jcp.27038

**Published:** 2018-08-04

**Authors:** Wang Chengji, Fan Xianjin

**Affiliations:** ^1^ College of Physical Education, Chaohu University Hefei Anhui China

**Keywords:** apoptosis, diabetic cardiomyopathy, endoplasmic reticulum stress (ERS), exercise training

## Abstract

To explore the protective effect of exercise training on the injury of myocardium tissues induced by streptozotocin (STZ) in diabetic rats and the relationship with endoplasmic reticulum stress (ERS), the male sprague‐dawley (SD) rats were fed with high‐fat and high‐sugar diet for 4 weeks, followed by intraperitoneal injection of STZ, 40 mg/kg, to establish a diabetes model, and then 10 rats were randomly selected as diabetes mellitus (DM) controls and 20 eligible diabetic rats were randomized into two groups: low‐intensity exercise training (*n* = 10) and high‐intensity exercise training (*n* = 10). After 12 weeks of exercise training, rats were killed and serum samples were used to determine cardiac troponin‐I (cTn‐I). Myocardial tissues were sampled for morphological analysis to detect myocardial cell apoptosis, and to analyze protein expression of glucose‐regulated protein 78 (GRP78), C/EBP homologous protein (CHOP), and caspase‐12. Different intensities (low and high) significantly reduced serum cTn‐I levels compared with the DCM group (*p* < 0.01), and significantly reduced the percentage of apoptotic myocardial cells and improved the parameters of cardiac function. Hematoxylin and eosin and Masson staining indicated that exercise training could attenuate myocardial apoptosis. Additionally, exercise training significantly reduced GRP78, CHOP, and cleaved caspase‐12 protein expression in an intensity‐dependent manner. These findings suggest that exercise appeared to ameliorate diabetic cardiomyopathy by inhibiting endoplasmic reticulum stress‐induced apoptosis in diabetic rats.

## 1 INTRODUCTION

In human and animal models of diabetes, a heart muscle‐specific disease in the absence of other vascular pathology has been described, termed as diabetic cardiomyopathy (Gilca et al., 2017; Lee & Kim, 2017). The pathophysiology of diabetic cardiomyopathy is not completely understood as several mechanisms can be involved, including oxidative stress, inflammation, myocardial fibrosis, endoplasmic reticulum (ER) stress, and apoptotic cell death. All of these have been proposed as potential contributing factors in the pathogenesis of diabetic cardiomyopathy (Miki, Yuda, Kouzu, & Miura, 2013).

The ER is a central organelle entrusted with lipid synthesis, protein folding, and protein maturation, and the ER is involved in the intrinsic pathway of apoptosis (Xu, Zhou, Xu, & Cai, 2012). Various conditions, including hypoxia, ischemia, elevated protein synthesis, exposure to free radicals, hyperhomocysteinemia, and gene mutations, can induce the pathological accumulation of unfolded proteins in the ER, a condition referred to as ER stress (Liu, Chen, & Chen, 2016; Szegezdi, Logue, Gorman, & Samali, 2006); this further activates the unfolding protein response (UPR). The UPR can be considered as a safeguard for protein synthesis, posttranslational modifications, folding and secretion, calcium storage and signaling, and lipid biosynthesis (Bravo et al., 2013; Guharoy, Bhowmick, Sallam, & Tompa, 2016; Harding, Calfon, Urano, Novoa, & Ron, 2002; Xu, Bailly‐Maitre, & Reed, 2005; Zhang & Kaufman, 2006).

One action of UPR is to activate the expression of glucose‐regulated protein 78 (Grp78), which binds/activates three distinct stress response pathways: protein kinase‐like ER kinase (PERK), inositol requiring kinase 1α (IRE1 α), and activating transcription factor 6 (ATF6). Moderate ER stress could alleviate injury triggered by stress, but severe and chronic stress could lead to apoptosis and induce many diseases. The activation of c‐Jun N‐terminal kinase (JNK) and the transcriptional induction of C/EBP homologous protein (CHOP) and caspase‐12‐dependent pathways could initiate apoptotic processes (Xu et al., 2011; Zhao & Ackerman, 2006). Recently, more studies have strongly demonstrated the critical role of ER stress in the development of diabetic cardiomyopathy. Experimental evidence suggests that two ER stress hallmarks, GRP78 and caspase‐12, were upregulated in the diabetic rat hearts compared with normal rat hearts (Li et al., 2007).

Exercise is one of the therapies for the treatment of obesity and type 2 diabetes (Lindström et al., 2006). Muscle contraction during exercise results in the production of reactive oxygen species (ROS) and a subsequent increase in the expression of antioxidant enzymes (McArdle, Pattwell, Vasilaki, Griffiths, & Jackson, 2001). This increase of ROS may cause ER stress, resulting in an increase in the production of cellular antioxidants to protect against cell damage. The therapeutic effects of exercise and the biological responses it induces are known to be closely related to the intensity of exercise (Hansen, Dendale, van Loon, & Meeusen, 2010). Moreover, these biological responses vary according to exercise intensity and therefore induce different degrees of ER stress. Thus, we decided to compare ER stress, apoptosis signaling in low‐ and high‐intensity exercise‐trained rats.

## 2 MATERIALS AND METHODS

### 2.1 Materials

Fifty adult male sprague‐dawley (SD) rats (200 ± 20 g) were purchased from the Animal Center of Fudan University (China). Rats were housed at 20–22°C on a 12‐hr light–dark cycle. Rats were separated into high‐fat and high‐sugar diet rats (*n* = 40) and control rats (Con; *n* = 10). The former were fed with high‐fat and high‐sugar diet for 4 weeks and then given intraperitoneal injection of streptozotocin twice (STZ at 40 mg/kg; Sigma‐Aldrich (Shanghai, China) dissolved in citrate buffer, pH 4.5), and the latter were fed with regular chow and injected with the same of citrate buffer. Five weeks after the STZ injections, blood samples were harvested from the rat tail vein after 12 hr of fasting. The levels of fasting blood glucose (FBG) were measured in spectrophotometry‐based assays using commercially available kits (Invitrogen). Those rats with FBG > 7.8 mmol/L were considered to be diabetic rats. The FBG of control rats is normal. STZ‐induced diabetic rats were randomly studied in the following three different treated groups: diabetic model (diabetic cardiomyopathy [DCM]; *n* = 10), low‐intensity training (LIT; *n* = 10), or high‐intensity training (HIT; *n* = 10).

Goat polyclonal anti‐GRP78, anti‐CHOP, and anti‐caspase‐12 were purchased from Shanghai RuiQi Biology Technology Co., Ltd., China.

### 2.2 Exercise training

Before beginning the exercise protocols, the rats of both exercise groups were adapted to running on a treadmill for 15 min, at increasing speeds from 0 to 15 m/min, once per day for 5 days. After this adaptation, the rats ran on treadmills with a 10^°^ incline for a period of 60 min on 5 days per study week. The treadmill speeds were set at 20 m/min in the LIT group and 34 m/min in the HIT group. This experiment was performed as described previously (Garekani, Mohebbi, Kraemer, & Fathi, 2011). The trained rats were killed 3 days after the final exercise session to exclude the possible effects of acute exercise stress in the analysis. The study was conducted in accordance with ethical standards in sports medicine and exercise science (Harriss, Macsween, & Atkinson, 2017).

### 2.3 Sample collection

After 12 weeks of exercise, all rats were killed under anesthesia attained with intraperitoneal injection of 2 ml of 3% pentobarbital sodium. Blood samples obtained from the abdominal aorta were then centrifuged at 2,059*g* for 10 min. The supernatant was collected for determination of cardiac troponin I (cTn‐I) levels by using the AU400 automatic Biochemical Analyzer (Olympus Co., Tokyo, Japan). After collecting the blood samples, rat hearts were dissected and washed with 0.9% cold saline; vessels and connective tissue around the heart, atrial, and right ventricular tissue were separated. The harvested cardiac tissue was cut into two parts: the apical tissues were immediately frozen in liquid nitrogen until western blot analysis; remaining tissues were fixed in 10% neutral buffered formalin.

### 2.4 Echocardiographic evaluation

Rat hearts were measured with echocardiography to compare the development of diabetic cardiomyopathy. Two‐dimensional and M‐mode echocardiography images of rats were obtained using a commercially available 12 MHz linear array transducer system and an echocardiogram machine. M‐mode recordings were of the left ventricle at the level of the mitral valve in the parasternal view using two‐dimensional echocardiography guidance in both the short‐ and long‐axis views. Pulsed Wave Doppler was used to examine mitral diastolic inflow in the apical four‐chamber view. For each measurement, the data were averaged from three consecutive cardiac cycles. All measurements were made from digital images captured at the time of the study by the use of inherent analysis software (Sonos 5500 software packages).

### 2.5 Histological evaluation

Isolated heart tissues were paraffin embedded and the tissue specimens were cut into 5‐μm sections for subsequent hematoxylin and eosin staining. Masson staining was used to detect interstitial fibrosis. Images were visualized by using a light microscope (Olympus BX51; Olympus Co.) and digital imaging system (Olympus DP71; Olympus Co.). The hematoxylin and eosin‐stained images were observed at 400× magnification; tissue sections stained with Masson stain were examined at 40× magnifications. The investigators involved in histological evaluation were unknown to the study group. Images of the stained sections were analyzed with Image‐Pro Plus 6.0 image analysis software (Media Cybernetics, Rockville, MD). The myocardial collagen volume fraction (CVF) was calculated as the collagen area/total area. We selected five visual fields of per section, took 15 sections from each group, and then calculated the mean CVF.

### 2.6 Western blot analysis

After extraction of myocardial proteins, equal amounts of the protein preparations were separated by 15% sodium dodecyl sulfate polyacrylamide gel electrophoresis (SDS‐PAGE), as described by Wang et al. (2009). The separated proteins were transferred to nitrocellulose membranes (Invitrogen) for 50 min at 120 V. The membrane was blocked with 5% non‐fat milk in phosphate buffered saline (PBST; pH 7.6, containing 0.05% Tween‐20) for 2 hr at room temperature and then incubated with a primary antibody against Grp78 (1: 500, Santa Cruz), caspase‐12 (1: 500, Sigma), CHOP (1: 500 and 1: 500, resp., Stressgen), and β‐actin (1: 500, Santa Cruz) at 4°C overnight. After incubating with 1: 4,000 horseradish‐peroxidase‐(HRP)‐conjugated anti‐mouse/rabbit/goat IgG (Santa Cruz), the blots were developed using enhanced chemiluminescence (PE Applied Biosystems). The membranes were scanned densitometrically by Typhoon (Pharmacia) and quantitated using Image Total Tech (Pharmacia).

### 2.7 Data analysis

All results were expressed as mean ± *SD*. Comparisons between study groups were made by one‐way analysis of variance, followed by Bonferroni's post hoc test. Data were analyzed with SPSS (version 17.0) software (SPSS Inc., Chicago, IL), and *p* < 0.05 was considered as statistically significant.

## 3 RESULTS

### 3.1 Effects of exercise on echocardiographic parameters

Echocardiographic parameters of cardiac function are summarized in Table 1. Compared with the Con group, left ventricular ejection fraction (LVEF), left ventricular end systolic volume (LVES), and the early (E) to late (A) ventricular filling velocities (E/A) were significantly lower and left ventricular end‐diastolic diameter (LVEDD) or left ventricular end diastolic volume (LVPWD) were significantly higher in the DCM group (all *p* < 0.05). HIT were associated with an improvement in LVEF, LVES, E/A, and LVEDD compared with the DCM group (*p* < 0.05). LIT was associated with a decreased LVPWD value compared with those in the DCM group (*p* < 0.05).

**Table 1:** Comparison of echocardiographic parameters after exercise

	Con	DCM	LIT	HIT
E/A	2.45 ± 0.99	0.32 ± 0.04^a^	0.31 ± 0.03^a^	0.35 ± 0.1^a^
EF (%)	88.25 ± 0.96	75.75 ± 4.57	74.56 ± 6.96	75.13 ± 2.95^b^
FS (%)	52.25 ± 0.96	44.5 ± 2.52	39.33 ± 8.46	38.88 ± 2.3^b^
LVPW (mm)	3.47 ± 0.4	4.53 ± 0.5	3.31 ± 0.38^c^	2.72 ± 0.31^cd^
LVEDD (mm)	5.54 ± 0.99	6.54 ± 0.35	5.47 ± 0.59^c^	5.13 ± 0.26^b^

*Note*. Con: control; HIT: high‐intensity exercise training; LIT: low‐intensity exercise training.

^a^Con group; ^b^LIT group; ^c^DCM group; ^d^LIT group.

### 3.2 Effects of exercise on serum cTn‐I levels

**Figure 1:**
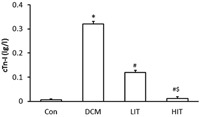
Effects of exercise on serum cTn‐I levels. Data are expressed as mean ± standard deviation. *p < 0.01 versus Con group; ^#^ *p* < 0.01 versus DCM group; ^&^ *p* < 0.01 versus LIT group. Con: control; HIT: high‐intensity exercise training; LIT: low‐intensity exercise training

As shown in Figure 1, serum cTn‐I levels in the diabetic model group were significantly higher than those in the Con group (*p* < 0.01). Compared with the diabetic model group, exercise training was associated with decreased cTn‐I levels (*p* < 0.01); there was a significant difference between the LIT and the HIT groups (*p* < 0.01).

### 3.3 Effects of exercise on myocardial pathological abnormalities

**Figure 2:**
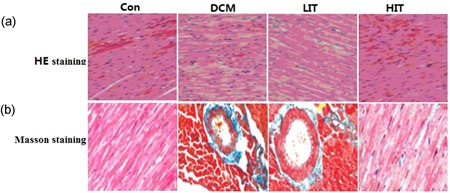
Effects of exercise training on histopathological abnormalities in myocardial tissue. (a) Hematoxylin and eosin (HE) staining (400× magnification) showed irregular arrangement of myocardial cells, uneven distribution of cytoplasm, and ruptured myocardial fibers in the DCM group, whereas exercise training attenuated the above histopathological changes. (b) Masson staining (40× magnification) showed irregular and noticeably increased interstitial collagen fibers (green region) in the DCM group; HIT significantly attenuated histopathological changes. Con: control; HIT: high‐intensity exercise training; LIT: low‐intensity exercise training [Color figure can be viewed at wileyonlinelibrary.com]

On hematoxylin and eosin staining (Figure 2a), myocardial cells in the Con group appeared to be more compact and arranged in an orderly manner, with bright red cytoplasm and centrally located oval nuclei. No dissolved muscle fibers or any evidence of vacuolar degeneration and mononuclear cell infiltration were observed. However, in the DCM group, disorderly arranged myocardial cells, uneven cytoplasm distribution, rupture of myocardial fibers, and irregular nuclei were visualized. Exercise training attenuated the above myocardial injury, particularly in rats that had high intensities of exercise training. As shown on Masson staining (Figure 2b), in the Con group, intact collagen fibers and no obvious myocardial interstitial collagen deposition were found. In the DCM group, myocardial cells showed an irregular arrangement and increased interstitial collagen fibers in the intercellular and perivascular space. Exercise training reduced the collagen fibers (green belt), particularly in high‐intensity exercise training rats.

### 3.4 Effect of exercise on myocardial cell apoptosis

**Figure 3:**
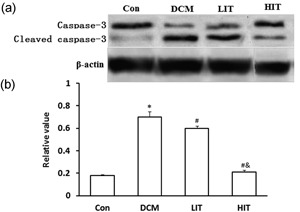
Effects of exercise training on the activation of caspase‐3 in the DCM and Con. (a) Western blot was performed using each relevant antibody. β‐Actin was shown as a loading control. (b) Statistical analysis. Data were shown as mean ± standard deviation. ^∗^ *p* < 0.05 versus Con rats, ^#^ *p* < 0.05 < 0.01 versus DCM rats; ^&^ *p* < 0.05 versus LIT rats. Con: control; HIT: high‐intensity exercise training; LIT: low‐intensity exercise training

The expression of caspase‐3 is measured to evaluate the apoptosis of cardiac myocyte because ER stress‐associated apoptosis is significantly associated with cardiac remodeling. Our western blotting showed that caspase‐3 was activated significantly in DCM rats (Figure 3; *p* < 0.05). However, caspase‐3 was significantly inhibited in the exercise training group, especially in the HIT group, which shows that exercise training can decrease the apoptosis of cardiomyocyte.

### 3.5 Exercise training decreases ER stress‐induced myocardial apoptosis by downregulating the expression of CHOP and Grp78 and Inactivating Caspase‐12

**Figure 4:**
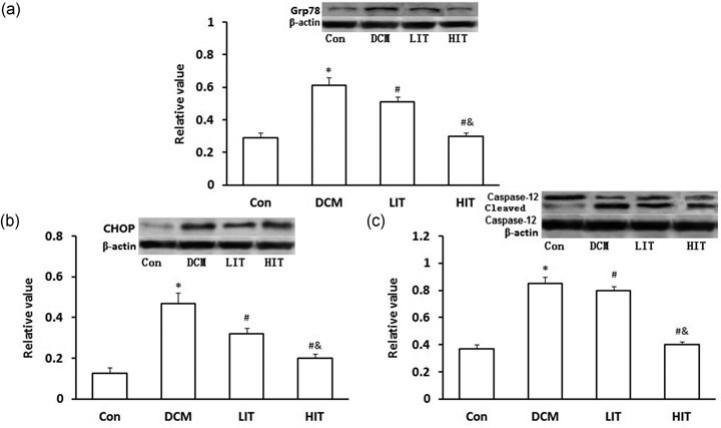
Immunoblot analysis for (a) Grp78, (b) CHOP, and (c) caspase‐12 in the myocardium of DCM and Con rats. The upper trace of each panel shows representative blots of proteins in DCM and Con rats. The lower panels show the bar graphs summarizing the immunoblot data. Western blot was performed using each relevant antibody. β‐Actin was shown as a loading control. Data were shown as mean ± *SD*. ^∗^ *p* < 0.05 versus Con rats, ^#^ *p* < 0.05 versus DCM rats, ^&^ *p* < 0.05 versus LIT rats. CHOP: C/EBP homologous protein; Con: control; HIT: high‐intensity exercise trainin; LIT: low‐intensity exercise training

Representative GRP78 band with a molecular weight of 78 kD of western blot analysis is shown in Figure 4a. The myocardial GRP78 protein level was increased in DCM rats compared with Con group rats (*p* < 0.01). Exercise training significantly decreased the myocardial GRP78 protein level compared with in the DCM rats (*p* < 0.01). Figure 4 b shows the representative CHOP band with a molecular weight of 29 kD on western blot analysis. An increased CHOP protein level was observed in DCM rats as compared with the normal controls (*p* < 0.01). Exercise training significantly decreased the CHOP protein level in the myocardial tissues as compared with that in the untreated diabetic rats (*p* < 0.01). Furthermore, the normal rat myocardium showed caspase‐12 band with a molecular weight of 40 kD, and a weak band with a molecular weight of 17 kD. An increase in cleaved caspase‐12 protein level was observed in untreated diabetic rats as compared with that in the normal controls (*p* < 0.05). Exercise training significantly reduced the cleaved caspase‐12 protein level in the myocardial tissues than those in the untreated diabetic rats (*p* < 0.05; Figure 4c).

## 4 DISCUSSION

The current study demonstrates that exercise training exerts cardioprotective effects in experimental models of diabetic cardiomyopathy. The results of histological examination of the hematoxylin and eosin and Masson‐stained showed disordered myocardial cells, rupture of myocardial fibers, and interstitial fibrosis in diabetic rat heart tissue. Diabetic cardiac hypertrophy was demonstrated by echocardiography. The occurrence of myocardial insult was substantiated by elevated cardiac enzyme levels. Our western blotting showed that caspase‐3 was activated significantly in DCM rats. However, caspase‐3 was significantly inhibited in the LIT and HIT groups, especially in the HIT group, which shows that exercise training can decrease the apoptosis of cardiomyocyte.

Exercise training reduced serum cTn‐I levels and the biochemical markers of myocardial cellular injury; improved parameters of cardiac function; decreased myocardial cell apoptosis; reduced GRP78, CHOP, and cleavage of caspase‐12 protein expression. The findings suggest that exercise attenuated diabetic myocardial damage in STZ‐induced diabetic rats through reducing the CHOP/caspase‐12‐induced apoptosis in ER stress.

Recent studies indicated that hyperglycemia‐caused ER stress played an important role in diabetic cardiomyopathy (Mulhern et al., 2006), which is consistent with our study. The ER plays an essential role in the modification process after protein synthesis and is also where the disposal of abnormally folded proteins begins. Normally, the UPR could result in upregulation of ER stress‐associated chaperone synthesis. GRP78 is a key regulator of the UPR such that it binds and maintains the transmembrane ER stress sensors (PERK, IRE1, and ATF6) in their inactive forms, and on ER stress, GRP78 is released resulting in the activation of these signaling pathways, impacting both cell survival and apoptosis (Bertolotti, Zhang, Hendershot, Harding, & Ron, 2000; Luo & Lee, 2013).

Actually, in our study, Grp78 is involved in ER stress in DCM rats and exercise training can inhibit expression of Grp78.

High‐intensity or long‐term exercise training increases the number and quality of mitochondria by effectively stimulating mitochondrial biogenesis (Holloszy, 2008). Although the reasons for this adaptation mechanism are still unclear, recent research has suggested that this phenomenon is caused by the exercise‐induced promotion of the transcription of mitochondrial proteins to consequently reduce the expression of UPR (Ogborn, McKay, Crane, Parise, & Tarnopolsky, 2014).

The underlying mechanism of diabetic cardiomyopathy is complex (Bugger & Abel, 2014). Multiple pathological stimuli including ischemia, oxidative stress, hypoxia, hyperglycemia, and hyperlipidemia are known to cause ER stress. However, exposure to high glucose levels appears to be the central mechanism of ER stress 30. ER stress‐related apoptotic signaling proteins include CHOP and caspase‐12.

CHOP is the downstream protein of the apoptotic pathway and plays an important role in ER stress‐induced apoptosis. CHOP can be activated by the overtranscription of ATF4, TRAF2, and XBP1 (Kim, Xu, & Reed, 2008; Oyadomari & Mori, 2004). Accumulation of CHOP can promote the transcription of ATF4, TRAF2, and XBP1, and overexpression of this factor can sensitize the ER stress of cells via increasing the expression of the CHOP protein. To explore the antidiabetic cardiomyopathy function of exercise training in DCM, CHOP is a key target. Our results showed that the expression of CHOP was significantly upregulated in DCM rats compared with Con rats. The overexpression of CHOP in DCM rats can be significantly inhibited by a exercise training, especially in high‐intensity exercise training, which shows a intensity effect of exercise on the inhibition of the CHOP expression.

Caspase‐12 is exclusively located at the ER, and, following its activation, it can directly process downstream caspases in the cytosol, mainly caspase‐9 and caspase‐3 (Liu & Baliga, 2005). Caspase‐12‐mediated apoptosis is a specific apoptosis pathway of the ER, and apoptosis that occurred because of membrane or mitochondrial targeted signals would not activate caspase‐12 (Ohse et al., 2006). In this study, caspase‐12 also participates in ER stress in the DCM rats through enhancing its activity. This activated caspase‐12 could only be blocked significantly by the high‐intensity exercise training, which showed the same intensity‐dependent effect as CHOP protein.

Of these three signaling proteins, caspase‐12 and CHOP are specific apoptotic pathways of ER. In this study, upregulation of GRP78, CHOP, and cleaved caspase‐12 protein expression was found in the diabetic myocardium, which indicates that excessive ER stress was involved in the pathology of diabetic cardiomyopathy. Treatment with exercise attenuated diabetic myocardial injury as well as decreased apoptosis in cardiocytes. In addition, exercise appeared to reduce GRP78 and CHOP, and cleaved caspase‐12 protein expression in an intensity‐dependent manner. Together these findings suggest that the cardioprotective effect of exercise may be mediated through the ER stress‐induced apoptosis. Therefore, downregulation of excessive cell apoptosis appears to play a dominant role in the cardioprotective mechanism.

## 5 CONCLUSIONS

In summary, exercise training improves diabetic cardiomyopathy and heart function by the decrease of cardiac myocyte ER stress and subsequent myocardial apoptosis. This is an important finding because ER stress is one of the underlying mechanisms of diabetic complications. Thus, exercise training may significantly contribute to complication prevention in diabetes, by showing an intensity effect. Our findings reveal a novel mechanism of effect of exercise in the management of diabetic cardiomyopathy.

## CONFLICTS OF INTEREST

All authors declare that there are no conflicts of interest.

## AUTHOR CONTRIBUTIONS

W.C. participated in the study design, analysis, report development, and interpretation of study findings. F.X. participated in writing the report.
